# ROS, an Important Plant Growth Regulator in Root Growth and Development: Functional Genes and Mechanism

**DOI:** 10.3390/biology13121033

**Published:** 2024-12-10

**Authors:** Jialin Su, Yumei Liu, Fengqing Han, Fuxin Gao, Fangyi Gan, Ke Huang, Zhansheng Li

**Affiliations:** 1 College of Horticulture, Hunan Agricultural University, Changsha 410128, China; sujialin007@163.com; 2 State Key Laboratory of Vegetable Biobreeding, Institute of Vegetables and Flowers, Chinese Academy of Agricultural Sciences, Beijing 100081, China

**Keywords:** ROS, plant root, growth and development, functional genes, regulation mechanism

## Abstract

Roots are fundamental to the growth, development, and survival of plants. Beyond anchoring the plant, roots absorb water and nutrients, supporting the plant’s ability to grow and function normally. Root systems, originating from the apical meristem, exhibit significant diversity depending on the plant species. ROS are byproducts of aerobic metabolism, present in both above- and below-ground plant tissues. While ROS were once considered merely harmful byproducts of oxygen metabolism, they are now recognized as critical signaling molecules that regulate plant growth and development. Under stress conditions, plants produce elevated levels of ROS, which can inhibit growth. However, moderate ROS levels act as signals that integrate various regulatory pathways, contributing to normal plant development. However, there is still a lack of comprehensive and systematic research on how ROS precisely regulate root growth and development. This review provides an overview of ROS production pathways and their regulatory mechanisms in plants, with a particular focus on their influence on root development.

## Simple Summary

Reactive oxygen species (ROS) are vital molecules for the growth and development of plants. Roots, as the primary organs for nutrient and water absorption, play a critical role in sustaining plant life. In this review, we explore the mechanisms of ROS production in plants, the developmental processes of plant roots, and how ROS influence root development. This synthesis aims to provide insights and guidance for future research on the interplay between ROS and root development.

## 1. Introduction

ROS are present in various forms in all aerobic organisms, including free radical species, such as superoxide anion (O_2_·^−^), hydroxyl radicals (·OH), peroxy radicals (ROO·), and alkoxy radicals (RO·). Additionally, ROS exist as non-radical molecules, including ozone (O_3_), singlet oxygen (^1^O_2_), and hydrogen peroxide (H_2_O_2_) [1,2,3]. Higher plants have evolved specialized pathways to use ROS as signaling molecules, regulating responses to biotic and abiotic stresses while mitigating oxidative damage [4,5,6,7,8]. When ROS concentrations are too high, they can cause damage to plants. However, at moderate levels, ROS serve as essential secondary messengers, regulating intracellular signal transduction and contributing to plant growth and development [1,9,10,11].

The root system architecture (RSA) of plants consists of the primary root (PR), lateral roots (LRs), root hairs (RHs), and other components. RSA development relies on a balance between root stem cell proliferation and differentiation [12]. Stem cells are located in the root apical meristem (RAM), which is divided into three zones: the meristematic, elongation, and maturation zones [13]. In the meristematic zone, undifferentiated stem cells divide to produce progenitor cells. These cells expand in the elongation zone and eventually mature in the maturation zone, differentiating into specialized root tissues. Together, cell proliferation and elongation drive root growth, shaping the plant’s ability to explore soil environments [12].

Since the emergence of oxygen-producing photosynthetic organisms around 2.2 to 2.7 billion years ago, ROS have been intrinsic to life on Earth [14]. Elevated ROS concentrations within cells cause oxidative stress, leading to damage to cellular membranes, proteins, RNA, and DNA. To counteract this, plants rely on a highly efficient ROS detoxification system composed of major ROS-scavenging enzymes, including superoxide dismutase (SOD), ascorbate peroxidase (APX), catalase (CAT), and glutathione peroxidase (GPX), along with antioxidants, such as ascorbic acid and glutathione. These components not only neutralize ROS like O_2_·^−^ and H_2_O_2_ but also regulate ROS as essential signaling molecules. The regulation of ROS dynamics is governed by a complex gene network known as the ROS gene network. This network coordinates ROS signaling with other cellular responses, including Ca^2+^ signaling. Calcium ion signals are stored in organelles and are rapidly released upon environmental stimuli, activating downstream signaling pathways. ROS and Ca^2+^ signaling pathways interact with each other to coordinate the cell’s stress response, enhancing the plant’s adaptability and defense capabilities [14,15,16,17].

There is evidence supporting the role of ROS in plant root development, particularly in the root apical meristem, root hair zone, and lateral root formation. ROS not only play an important role in cell proliferation and differentiation but also interact in complex ways with other signaling molecules, such as plant hormones. However, how ROS regulate plant growth in these processes remains unclear, and the interaction between ROS and root stress response pathways still lacks in-depth mechanistic studies.

## 2. ROS Production and Clearance

### 2.1. Photosynthesis

**Figure 1:**
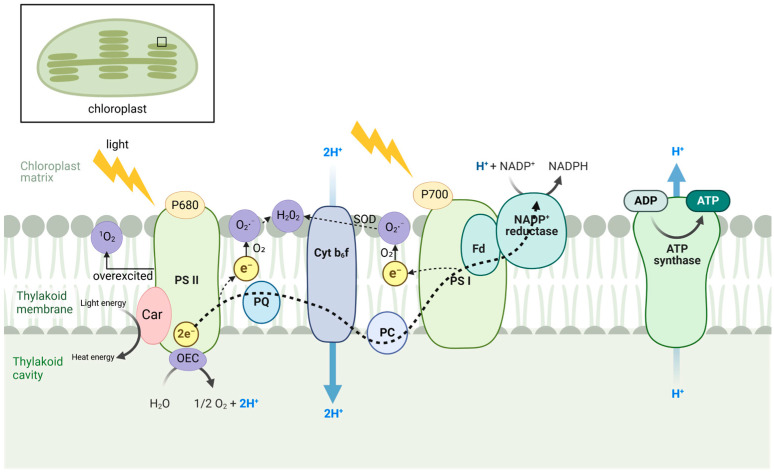
ROS generation in chloroplasts during photosynthesis. Image created with BioRender.com, with permission.

In plants, the light reactions of photosynthesis are a key source of ROS, mainly produced in photosystem I (PSI) and photosystem II (PSII), located in the thylakoid membranes (Figure 1) [18,19]. PSI absorbs light energy, exciting chlorophyll molecules and transferring the excited electrons through the electron transport chain to NADP+, thereby reducing it to form NADPH. PSI also participates in the Mehler reaction, transferring electrons to oxygen molecules and thereby generating O_2_·^−^, which can be converted into H_2_O_2_ [19]. PSII absorbs light to split water into oxygen, protons, and electrons, a process called water photolysis. Under intense light, PSII may become overexcited, causing P680 chlorophyll to enter a triplet state (^3^Chl) and interact with oxygen, forming singlet oxygen (^1^O_2_) [20]. In normal conditions, the electron transport chain in PSII transfers electrons to plastoquinone, providing chemical energy [21]. However, under stress, overloaded electron flow can cause electron leakage, generating O_2_·^−^ [22]. PSI and PSII are essential components of the photosynthetic electron transport (PET) system [23]. NADP^+^ reductase receives electrons from PSI to form NADPH, which powers carbon fixation. ATP synthase uses the proton gradient formed during electron transport to produce ATP. A key protective mechanism in PET is non-photochemical quenching (NPQ), which dissipates excess light energy as heat, reducing ROS production. Carotenoids help by absorbing excess light and converting it into heat, preventing excessive ROS generation [24,25].

### 2.2. Respiration

**Figure 2:**
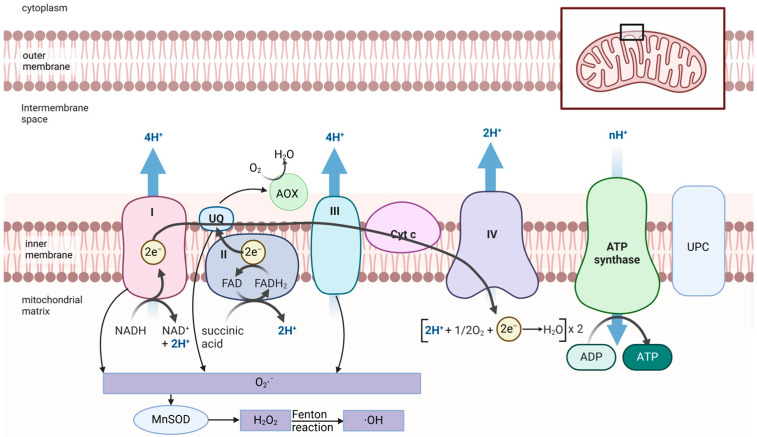
ROS generation in mitochondria during cellular respiration. Image created with BioRender.com, with permission.

In plant cells, the primary sources of ROS are chloroplasts and peroxisomes. However, in non-green tissues or under dark conditions, mitochondria become the dominant site of ROS production, mainly through the electron transport chain (ETC) (Figure 2) [26]. Complex I (NADH dehydrogenase) and Complex III (ubiquinone-cytochrome c oxidoreductase) are the primary ROS sources, where single electrons leak and reduce molecular oxygen to form O_2_·^−^. Complex II (succinate dehydrogenase) can also significantly increase ROS production via reverse electron transport (RET) under stress conditions such as ubiquinone pool over-reduction. These O_2_·^−^ are rapidly converted to H_2_O_2_ by mitochondrial manganese superoxide dismutase (MnSOD) [27]. However, excessive H_2_O_2_ accumulation may lead to the formation of highly toxic hydroxyl radicals through the Fenton reaction [28]. Plant mitochondria possess specific mechanisms, such as alternative oxidase (AOX) and uncoupling proteins (UCPs), to mitigate ROS generation and accumulation. AOX reduces molecular oxygen directly to water by accepting electrons from the ubiquinone pool, bypassing Complexes III and IV, thereby lowering the reductive pressure on the electron transport chain and reducing ROS production. UCPs, by leaking protons across the inner membrane, reduce excessive electron transport chain activity, significantly suppressing ROS generation. These mechanisms collectively regulate mitochondrial ROS production, ensuring redox homeostasis in plant cells [29,30].

### 2.3. Enzymatic Reactions

Enzymatic reactions in plants are crucial for both the production and scavenging of ROS. These enzymes help maintain ROS balance, generating ROS as signaling molecules while eliminating excess ROS to prevent oxidative damage. NADPH oxidase (RBOH), a transmembrane enzyme located in the plasma and chloroplast membranes, catalyzes the transfer of electrons from NADPH to oxygen, producing O_2_·^−^. RBOH plays a key role in signal transduction during plant responses to hormones (e.g., abscisic acid) and environmental stresses, such as drought, salinity, and pathogen attacks. Its activity is tightly regulated by Ca^2+^ and phosphorylation to ensure precise ROS production and prevent cellular damage [31,32,33]. Xanthine oxidase (XO), involved in purine metabolism, oxidizes hypoxanthine and xanthine to uric acid, generating ROS such as H_2_O_2_. Uric acid itself has antioxidant properties, meaning that XO plays a dual role in plant defense by both producing and scavenging ROS [34,35].

SOD is the first line of defense in the plant antioxidant system, converting O_2_·^−^ into H_2_O_2_ and O_2_, thereby reducing the toxicity of O_2_·^−^. Class III peroxidases collaborate with SOD to regulate ROS dynamics by decomposing H_2_O_2_. However, in the presence of iron, H_2_O_2_ can participate in the Fenton reaction under stress or iron overload, producing highly reactive hydroxyl radicals [28,32]. CAT is a key enzyme for breaking down H_2_O_2_ into water and oxygen. Primarily found in peroxisomes and mitochondria, CAT plays an important role in photorespiration and β-oxidation processes [36]. APX reduces H_2_O_2_ to water using ascorbate as an electron donor. The ascorbate-glutathione cycle, involving glutathione reductase (GR) and glutathione peroxidase (GPX), maintains reduced glutathione (GSH) levels to scavenge ROS and prevent lipid peroxidation. APX and GR/GPX functions are interdependent, working together through the ascorbate-glutathione cycle to protect plant cells from oxidative damage [37].

Plant-specific enzymes also play a critical role in regulating ROS dynamics. Lipoxygenase (LOX) catalyzes fatty acid peroxidation and amplifies ^1^O_2_ production through Russell reactions under stress conditions. LOX is involved in signal transduction and defense mechanisms, including modulating root growth during osmotic stress by generating ROS such as ^1^O_2_ and potentially contributing to oxidative signaling [38]. The cytochrome P450 enzyme system, essential in secondary metabolism, produces ROS like H_2_O_2_ as byproducts during the synthesis of alkaloids, terpenoids, and flavonoids. These ROS act as intermediates and signaling molecules, regulating cellular functions and responses to external stimuli [39]. Nitric oxide (NO) is another critical component of the ROS network in plants. Although plants do not possess the classical nitric oxide synthase (NOS), they synthesize nitric oxide (NO) through a reductive pathway involving nitrate reductase (NR) and nitrite reductase (NiR). This process helps maintain a balance between NO and ROS signaling, which is essential for plant growth, development, and stress tolerance [40].

## 3. Root Growth and Development

### 3.1. Root System Architecture

**Figure 3:**
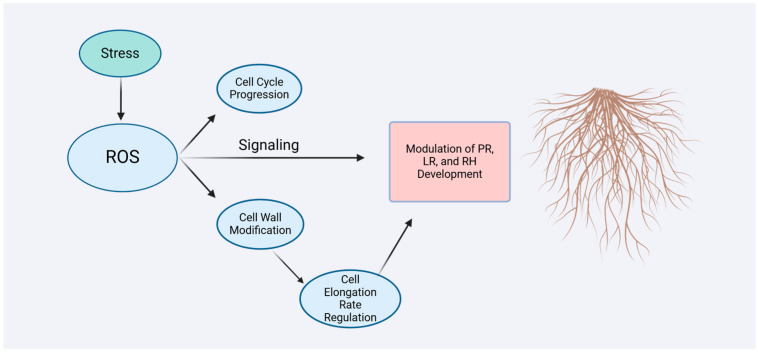
Schematic diagram of major pathways through which ROS affects root system architecture. Image created with BioRender.com, with permission.

ROS regulates root growth and morphology by modulating cell division, expansion, hormonal signaling pathways, and cell wall remodeling (Figure 3). Additionally, ROS is involved in plant responses to environmental stresses, regulating root architecture to adapt to unfavorable growth conditions. It plays a crucial role in root development and adaptive adjustments. Investigation of RSA involves analyzing a range of polygenic traits that characterize sub-root system features, including primary root length, total root length, root angle, root number, root diameter, root length density, root growth pattern, and root surface area [41]. The regulation of RSA is a complex process governed by interactions among gasotransmitters, plant hormones, nutrients, and environmental factors. RSA plays a pivotal role in plant adaptation to environmental stresses, such as drought, salinity, and nutrient deficiencies [42]. Gasotransmitters, such as ammonia (NH_3_), NO, and hydrogen sulfide (H_2_S), play an important role in this process [43,44]. Studies demonstrate that roots adapt their morphology and structure under stress conditions by elongating, increasing branching, or adjusting the root-to-shoot ratio to optimize water and nutrient uptake. The plasticity of RSA enables plants to modify their growth strategies in response to environmental fluctuations, enhancing survival and productivity [45]. Moreover, genetic and molecular studies have identified key genes and signaling pathways involved in regulating RSA, providing a foundation for breeding and biotechnological innovations. This highlights RSA not only as a crucial component of plant physiological adaptation but also as an essential focus for achieving sustainable agricultural development in the future [46].

### 3.2. Regulation and Signaling Mechanisms of Stem Cells in Root Apical Meristems

**Figure 4:**
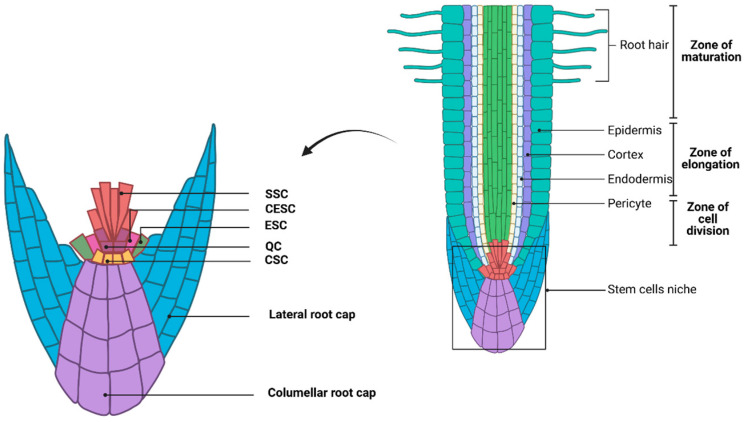
Plant root system structure and stem cell ecological niche. SSC: stele stem cell. CESC: endodermis–cortical stem cells. ESC: Epidermo-lateral cap stem cells. QC: quiescent center. CSC: columella stem cell. Image created with BioRender.com, with permission.

In plants, the root stem cell niche consists of a mitotically inactive quiescent center (QC) surrounded by a group of stem cells (Figure 4) [47]. The QC maintains the undifferentiated state of stem cells by preventing differentiation, ensuring continuous root growth [48,49]. The *WUSCHEL-LIKE HOMEOBOX5* (*WOX5*) gene is specifically expressed in the QC, where it regulates the expression of genes such as *CYCD3;3* to inhibit excessive cell division and maintain the quiescent state of the QC. *CYCD3;3* and *CYCD1;1* promote cell division, but their overexpression in *wox5* mutants leads to excessive cell division, disrupting QC function [13,50,51,52]. The interaction of WOX5 with its target gene, the transcription factor BRASSINOSTEROIDS AT VASCULAR AND ORGANIZING CENTER (BRAVO), restricts the mobility of WOX5, resulting in the self-confinement of *BRAVO* expression to the QC, where it also promotes QC quiescence [53].

The peptide CLE40 is secreted by differentiated columnar cells and indirectly regulates the expression of WOX5 through the signal transduction of receptor kinases ACR4 and CLV1, thereby regulating root development. This feedback loop balances cell proliferation and differentiation in the root meristem. The level of CLE40 affects WOX5 activity in a dose-dependent manner: low levels maintain the quiescent state of the QC, while higher levels restrict WOX5 expression to the QC, promoting the differentiation of columnar stem cells [51,54]. PLT1 and PLT2, members of the PLETHORA (PLT) family of AP2 domain transcription factors, form a protein concentration gradient in the root apex. At high concentrations in the QC, they maintain stem cell undifferentiation, while lower concentrations promote stem cell differentiation [55,56,57]. PLT proteins, in combination with *WOX5* and *CYCD*, regulate stem cell dynamics. Elevated PLT levels activate *miR396*, which degrades *GRF* mRNA, creating a negative feedback loop to prevent excessive proliferation [58]. *RGF1* (*ROOT GROWTH FACTOR 1*) activates the MAPK signaling pathway through its receptor *RGI1*, leading to the activation of MKK4/MKK5 and MPK3/MPK6, which ultimately regulate the expression of the transcription factors PLT1 and PLT2, thereby maintaining the function of the root apical meristem [59]. Additionally, exogenous RGF/GLV can promote the expansion of the root apical meristem, and mutants of the RGF/GLV receptors exhibit defects in meristem function, highlighting the importance of the RGF/GLV signaling in root development [60,61,62]. In root tip meristematic tissues, *SCARECROW* (*SCR*) and *SHORT ROOT* (*SHR*) play critical roles in endodermis and mesodermal sheath differentiation [63]. *SHR*, a mobile GRAS family transcription factor, originates in the central cylinder and migrates to the endodermal layer, where it activates *SCR* expression [64]. Together, SHR and SCR co-activate *CYCD6;1*, promoting endodermal cell division. In *shr* and *scr* mutants, the endodermal and pericycle sheaths are poorly developed [65,66,67].

### 3.3. Hormone Regulation

Auxin is a key regulator of root development, playing a crucial role in root growth. Its concentration is high at the root tip, promoting cell division, while it is lower in areas farther from the tip, where it helps maintain cell differentiation and development. Furthermore, auxin activates downstream signaling pathways, such as the RIT1-AUX/IAA-ARF pathway, by binding to receptors, which in turn regulate the expression of related genes [68,69]. Cytokinin precisely regulates root development through spatiotemporal signaling, ensuring proper function at different developmental stages. It promotes the transition from the elongation zone to the differentiation zone, where cells stop elongating and enter differentiation, thereby regulating growth cessation in the root [70]. Additionally, cytokinin enhances cell wall rigidity and works in conjunction with *AUX1* to further promote growth cessation. It also regulates the formation of cell identities in the protophloem and protoxylem, ensuring proper root development [70,71]. Abscisic acid (ABA) plays a crucial role in helping plants respond to environmental stresses and directly regulating root development. High concentrations of ABA inhibit cell division in the apical meristem and repress cell expansion in the root elongation zone. These effects are primarily mediated through the ABA signaling pathway. ABA also regulates root growth by limiting excessive elongation under normal conditions. During stress, ABA modulates water uptake by adjusting the osmotic pressure of root cells, either promoting or inhibiting water absorption depending on the situation [72,73]. Ethylene inhibits root elongation while promoting root thickening, lateral root formation, adventitious root formation, and root hair development. It modulates the expression of cell wall-loosening proteins, such as expansins, thereby restricting root cell elongation. At lower concentrations, ethylene, or its precursor ACC (1-aminocyclopropane-1-carboxylic acid), can promote root elongation [74]. Brassinosteroids (BRs) are a group of plant steroid hormones that play a crucial role in plant growth, development, and stress responses. They regulate the division and differentiation of stem cells in the root apical meristem, ensuring continuous root growth [75,76].

## 4. The Role of ROS in Root Growth and Development

### 4.1. Plant ROS Sensing and Signaling

ROS play a crucial role in plants by sensing abiotic and biotic stresses, integrating environmental signals, and activating stress response networks to enhance plant resilience. ROS act as signaling molecules and second messengers in plant biology, triggering downstream cascades that regulate processes like stomatal closure [77], programmed cell death [36], gravitropism [78], pollen–stigma interactions [79], and tolerance to biotic and abiotic stresses [80]. They also diffuse to various cellular compartments, causing oxidative post-translational modifications (Ox-PTMs) in proteins, including those involved in the Calvin cycle, transcription factors, and kinases [2,81,82]. A key ROS sensing mechanism involves the HYDROGEN PEROXIDE-INDUCED Ca^2+^ INCREASES 1 (HPCA1) sensor, a cell surface receptor kinase that recognizes H_2_O_2_, activating Ca^2^⁺ influx and downstream signaling, such as the MAPK pathway and defense-related gene expression [17,83,84,85]. ROS regulate several kinase pathways, including the MAPK, RBOH, and CDPK pathways, contributing to plant defense. Other genes involved in ROS generation, clearance, and regulation are listed in Table 1 [86,87,88,89].

In plant cells, ROS signaling pathways are regulated through extrinsic, intrinsic, and organellar routes [4]. Extrinsic ROS signaling involves enzymes in the cell wall and plasma membrane, such as RBOHs, aquaporins (AQPs), and peroxidases. RBOHs generate O_2_·^−^ in response to environmental stimuli in the apoplast. O_2_·^−^ then undergoes dismutation into H_2_O_2_ by SOD, and H_2_O_2_ can diffuse across the membrane to act as an early signaling molecule. Their activity is regulated by calcium ions, which modulate RBOH function, and protein phosphorylation, which can enhance or inhibit RBOH activity [82,90,91,92]. Intrinsic ROS signaling primarily involves antioxidant enzymes in the cytosol and organelles, such as SODs, catalases, and peroxidases, which regulate ROS levels and maintain redox homeostasis. Intrinsic signaling is also linked to pathways like the MAPK cascade and small GTPases (e.g., ROP/RAC), integrating ROS signals with other signaling molecules to coordinate the cellular response to oxidative stress, often triggered by metabolic processes. These pathways help the cell adapt to changes in its redox state during stress [93,94,95].

### 4.2. Regulation Mechanisms of ROS on Plant Root Stem Cell

ROS are crucial for stem cell development in the RAM. They regulate root growth through interactions with NO and hormones like auxin, cytokinins, and ethylene while maintaining stem cell niche (SCN) stability by affecting the cell cycle. O_2_·^−^ accumulates in the RAM and regulates SCN function [96,97]. PROHIBITIN3 (PHB3) helps maintain SCN integrity by limiting the expression of *ETHYLENE RESPONSE FACTORS* (*ERFs*), such as ERF115, ERF114, and ERF109. In *PHB3* mutants, the expression of QC markers like *WOX5* is reduced, highlighting *PHB3*′s role in SCN stability. *PHB3* also regulates ROS distribution in the root to stabilize the SCN [98,99]. The *APP1* gene, which regulates ROS homeostasis, promotes stem cell development by controlling ROS levels. Loss of *APP1* reduces ROS and promotes QC cells, while overexpression leads to ROS accumulation and SCN defects [100,101]. Recent research has also highlighted *XAL1*′s role in ROS signaling, particularly in primary root growth and stem cell differentiation under oxidative stress [102]. *AtSYP81* regulates ROS balance in the RAM, stabilizing SCN dynamics [103]. In the ABA hypersensitive mutant, excessive ROS impairs SCN function by reducing *PLT1* and *PLT2* expression [104]. BRs generate H_2_O_2_ in the SCN, modifying BZR1 and BES1 to promote root meristem formation and stabilize SCN function [105,106,107,108].

In ROS signaling, transcriptional regulation plays a key role in root development. *UPBEAT1* (*UPB1*), a basic helix–loop–helix (bHLH) transcription factor, regulates the expression of class III peroxidases in the elongation zone to maintain ROS balance [109]. *RGF1* controls the size of the root meristem through ROS signaling and enhances the stability of *PLT1* and *PLT2*. The *RGF1* receptor pathway regulates ROS distribution during meristem development, ensuring an optimal ROS gradient for stem cell niche maintenance [60,110]. Members of the *AP2/ERF* family are involved in plant responses to oxidative stress, disease resistance, and drought tolerance and are regulated by ROS-mediated signaling pathways [111]. ROS accumulation activates *WRKY* transcription factors, which regulate ROS-related genes to help plants cope with oxidative stress. This complex transcriptional network ensures proper root meristem function, balancing cell proliferation and differentiation to maintain root growth and development.

### 4.3. Effect of ROS on Root Elongation

ROS play a crucial role in modifying cell wall functions [112,113,114]. The primary cell wall (PCW) is composed of cellulose, pectin, hemicellulose, proteins, and water, and it plays a crucial role in supporting cell expansion and growth, thereby facilitating the development of plant tissues such as roots. The plasticity of the PCW is regulated by the remodeling of polysaccharides like pectin and hemicellulose. Reactive oxygen species (ROS) influence the activity of various enzymes, such as peroxidases and lignin biosynthesis enzymes, modulating the cross-linking and structure of cell wall components, which in turn affects the rigidity, plasticity, and extensibility of the cell wall [115,116]. Subsequently, the expansion process is achieved through the combined effects of osmosis, water absorption, and cell wall remodeling [117,118]. Peroxidases (PRXs) are enzymes that catalyze oxidation reactions using H_2_O_2_ as an electron acceptor, playing a key role in the structural remodeling of the cell wall [119]. They regulate the cross-linking reactions of lignin and polysaccharides (such as hemicellulose and pectin), thereby enhancing the mechanical strength and stability of the cell wall. Additionally, PRXs modulate the levels of ROS, such as hydrogen peroxide, to promote the cross-linking and regulation of cell wall components, thereby aiding plants in improving their resilience against environmental stresses and pathogen invasions [118,120,121,122,123].

Exogenous H_2_O_2_ treatment inhibits cell cycle-related gene expression and reduces root meristem size. It also affects cortical cell proliferation [67,124]. ROS regulate the transition of root cells from the division zone to the elongation and differentiation zones, with H_2_O_2_ crucial for meristem stability and influencing auxin synthesis, transport, and signaling [125]. O_2_·^−^ mainly accumulates in the extracellular matrix of the elongation zone, while H_2_O_2_ is concentrated in the cell wall of the differentiation zone and during root hair formation [121]. Disrupting this balance can affect root meristem size and development.

Lateral roots are formed from the existing primary roots, unlike adventitious roots that emerge from other parts of the plant. In early plant evolution, lateral roots were absent. Instead, they evolved as a mechanism to acquire more nutrients and adapt to different soil environments [126]. Auxin is a key hormone that regulates the formation of different types of roots [125,127]. Studies have shown that ROS regulate the development of lateral roots by modulating plant hormone signaling pathways, such as those involving ABA and auxin, and by upregulating genes like *LBD16*, which is involved in lateral root initiation, and *RGIs*, which contribute to root development [128]. In *Arabidopsis*, the NADPH oxidase protein AtrbohD1/F1 controls the production of superoxide in an auxin-independent manner, thereby negatively regulating lateral root formation [129]. After lateral root formation, a new QC is established, exhibiting a meristematic organization similar to that of the primary root. The subsequent development of lateral roots is similarly influenced by ROS, as observed in primary roots [130,131].

### 4.4. Root Hair Development Regulated by ROS

Root hairs are tubular structures formed by root epidermal cells that increase the surface area for nutrient absorption, interact with microorganisms, and help anchor the plant. During early development, ROS act as signaling molecules promoting the differentiation of root hair precursor cells. In *Arabidopsis*, ROS accumulate in root hair protrusions and migrate towards the tips as the hairs grow, ceasing when growth halts [132]. ROS generation is regulated by acid-sensitive ion channels, triggering signaling cascades that involve cell wall changes, cell polarity, and membrane reorganization, leading to root hair formation [133]. The balanced regulation of ROS is crucial for root hair length, morphology, and number, with both excessive and insufficient ROS causing abnormal development. ROS modulate cell division and elongation in root hairs through interactions with hormones and Ca^2^⁺ [134,135]. ROS also help root hairs adapt to environmental stresses like drought and salinity by activating antioxidant enzymes, enhancing stress resistance, and improving nutrient absorption and utilization [134]. Furthermore, ROS have been shown to affect root gravity response [78] and adventitious root formation [136].

**Table 1:** Genes or proteins that affect ROS in plants.

Phases	Gene/Protein Names	Functional Evidences	References
ROS generation	RBOHF RBOHD	RBOHF works with RBOHD in ROS-dependent ABA signaling in guard cells, and both are essential for the accumulation of reactive oxygen intermediates in plant defense.	[137,138,139]
	RBOHB	Overexpressing RBOHB significantly boosted ROS production, root nodule formation, and nitrogen-fixing activity. Additionally, an increase in the density of symbionts within root nodules was observed, which is crucial for plant growth and development.	[140,141]
	RBOHE	In RBOHE-deficient mutants, the loss of RBOHE function in the cortex and epidermis delayed lateral root emergence, reduced ROS in anthers, caused programmed cell death in the chorioallantois layer, and impaired pollen viability.	[31,142]
	RBOHC	The regulation of ROS production by Ca^2+^ channel activation is a key mechanism underlying the abortion of a significant proportion of pollen grains in RBOHC mutants. This process exerts a profound impact on pollen development and, consequently, on fertility.	[143,144]
ROS clearance	SOD	Protects cells from oxidative stress by converting harmful superoxide radicals into oxygen and hydrogen peroxide.	[145]
	CAT	Removes H_2_O_2_ and transforms it into H_2_O and O_2_.	[146]
	GPX	The catalyst facilitates the reduction of hydrogen peroxide and organic peroxides, converting peroxide into non-toxic substances and oxidizing glutathione to glutathione disulfide.	[147,148]
	GR	Reduction of glutathione disulfide (GSSG) to GSH.	[148]
	APX	A pathway for hydrogen peroxide removal by plants. Reduction of hydrogen peroxide to H_2_O and dehydroascorbic acid (DHA).	[90]
	Monodehydroascorbate reductase (MDHAR)	MDHAR is responsible for regenerating ascorbic acid from short-lived MDHA using NADPH as a reducing agent.	[15]
	Dehydroascorbate reductase (DHAR)	Reduction of DHA to ascorbic acid using reduced GSH as an electron donor.	[149]
	Thioredoxin reductase (TRX)	Target protein disulfides can be reduced to terminate signaling and maintain intracellular oxidant and reductant balance.	[90]
	*PHB3*	Both hydrogen peroxide and superoxide over-accumulated in root meristematic tissues of *PHB3*-deficient mutants.	[98]
ROS transcriptional regulation	*UPB1*	*UPB1* encodes a bHLH transcription factor that regulates root meristem organization by modulating the ROS gradient in roots.	[109]
	*AP2/ERF* transcription factor family	ERF115, ERF114, and ERF109 mediate ROS signaling in *Arabidopsis thaliana* to control root stem cell ecological niche maintenance and root growth via the phytosulfokine (PSK) peptide hormone.	[98]
	*WRKY* transcription factor family	WRKY79′s enhanced ROS scavenging and antioxidant activity reduced hydrogen peroxide and malondialdehyde levels in transgenic plants. WRKY40 helps plants cope with salt stress and accumulate organic acids by activating PbVHA-B1 expression and maintaining ROS balance.	[150,151,152,153]
	*NAC* transcription factor family	Overexpression of NAC089 promotes PCD by regulating PCD-related genes (e.g., NAC094), leading to increased caspase 3/7-like activity and ROS accumulation. ANAC013 and ANAC017 are involved in retrograde mitochondrial signaling and are believed to inhibit PCD by maintaining ROS homeostasis.	[154,155]
	AOX	Maintains redox balance during mitochondrial electron transfer and limits the formation of ROS, especially during stress.	[156]
	*LIKE SEX FOUR 2* (*LSF2*)	LSF2 regulates ROS homeostasis in response to oxidative stress, thereby controlling root development. *lsf2-1* mutants show reduced superoxide production and higher hydrogen peroxide levels in response to oxidative stress treatments compared to wild-type *Arabidopsis.*	[157]
	*ROOT HAIR DEFECTIVE SIX LIKE4* (*RSL4*)	Controlled by ARFs to induce local ROS synthesis. Involved in the regulation of root hair elongation and has target genes that promote root hair growth.	[158,159]
	FERONIA (FER)	The FER–LLG1 complex binds to ROP-GEF, which activates the RAC/ROPs plant RHO GTPase, leading to the activation of the NADPH oxidase RBOH and a subsequent localized burst of ROS.	[160]
	ZINC FINGER OF ARABIDOPSIS THALIANA12 (ZAT12)	Transcriptional profiling of ZAT12-overexpressing plants and wild-type plants under H_2_O_2_ stress showed that constitutive expression of ZAT12 in *Arabidopsis* resulted in enhanced expression of oxidative and light stress response transcripts.	[161]
	MAPK	RBOH, in conjunction with the MAPK pathway, integrates ROS signaling and regulates intercellular signal propagation in both local and systemic signaling, with the MAPK pathway playing a key role in converting ROS signaling into protein phosphorylation.	[86]

## 5. Challenges and Perspectives

This review outlines the significant role of ROS in plant development, particularly in root growth. ROS regulate various processes in root development, including cell division, cell wall loosening, and cell lysis, which are crucial for root formation, growth, and stress responses. ROS not only act directly on root cells but also indirectly modulate overall plant morphology through signaling networks, making them essential regulators of plant morphogenesis. However, the regulation of ROS levels must remain within an optimal range, as both excess and deficiency of ROS can negatively impact root development. This review will also explore the mechanisms of ROS generation, regulation, and signaling pathways, particularly their roles in roots. Additionally, the interactions between ROS, other organisms, and the environment will be analyzed, which is vital for a deeper understanding of root development in plants.

With the rapid advancement of high-throughput sequencing technologies, vast amounts of multi-dimensional data have been accumulated in the fields of plant genomics, transcriptomics, and proteomics. By integrating big data analytics with artificial intelligence (AI) technologies, these datasets can be efficiently mined and deeply analyzed, allowing for the identification of key genes closely associated with ROS signaling and root development. Specifically, the application of AI algorithms in clustering analysis and pattern recognition of gene expression profiles enables researchers to swiftly identify gene clusters related to ROS response and root developmental functions. The gene screening results driven by such data can provide critical insights for plant breeding. In modern breeding technologies, the application of molecular marker-assisted selection (MAS) and genomic selection (GS) methods facilitates the rapid introgression of beneficial genes into target crop varieties, enabling the development of new varieties with superior root system traits. Moreover, the precise manipulation of ROS and root development-related genes through CRISPR/Cas9 gene editing technology further enhances the ability to achieve breeding objectives with greater accuracy. The combination of these technologies holds significant potential for improving crop stress tolerance and promoting sustainable agriculture, offering novel approaches and methods for future plant breeding and agricultural production. Looking ahead, as research deepens and technology continues to evolve, the role of ROS in plant growth, development, and stress response will become increasingly well defined, and its application prospects will expand accordingly.

## 6. Conclusions

This review highlights the important role of ROS in regulating root growth and development in plants. While ROS are often seen as harmful byproducts, recent research shows they are crucial signaling molecules that help control processes like cell division, differentiation, and elongation in roots. ROS help balance growth and stress responses in roots by regulating enzymes and signaling pathways. The interaction between ROS and antioxidant systems ensures proper root function under different conditions. Additionally, understanding the genes involved in ROS production and detoxification could lead to genetic improvements in crops, such as better root development, nutrient uptake, and stress tolerance.

In conclusion, ROS play a key role in root development. Further research into ROS generation and the genes that regulate these mechanisms is crucial for advancing our understanding of plant growth, as well as improving crop productivity and resilience.

## Data Availability

The data presented in this study are available on request from the corresponding author.
